# Hydrophobically Modified siRNAs Silence Huntingtin mRNA in Primary Neurons and Mouse Brain

**DOI:** 10.1038/mtna.2015.38

**Published:** 2015-12-01

**Authors:** Julia F Alterman, Lauren M Hall, Andrew H Coles, Matthew R Hassler, Marie-Cecile Didiot, Kathryn Chase, Jasmin Abraham, Emily Sottosanti, Emily Johnson, Ellen Sapp, Maire F Osborn, Marian Difiglia, Neil Aronin, Anastasia Khvorova

**Affiliations:** 1 RNA Therapeutics Institute, University of Massachusetts Medical School, Worcester, Massachusetts, USA; 2 Department of Molecular Medicine, University of Massachusetts Medical School, Worcester, Massachusetts, USA; 3 Department of Medicine, University of Massachusetts Medical School, Worcester, Massachusetts, USA; 4 Department of Neurology, Mass General Institute for Neurodegenerative Disease, Charlestown, Massachusetts, USA

**Keywords:** delivery, Huntington's disease, neurodegenerative disease, RNA interference, small interfering RNA

## Abstract

Applications of RNA interference for neuroscience research have been limited by a lack of simple and efficient methods to deliver oligonucleotides to primary neurons in culture and to the brain. Here, we show that primary neurons rapidly internalize hydrophobically modified siRNAs (hsiRNAs) added directly to the culture medium without lipid formulation. We identify functional hsiRNAs targeting the mRNA of huntingtin, the mutation of which is responsible for Huntington's disease, and show that direct uptake in neurons induces potent and specific silencing *in vitro*. Moreover, a single injection of unformulated hsiRNA into mouse brain silences *Htt* mRNA with minimal neuronal toxicity. Thus, hsiRNAs embody a class of therapeutic oligonucleotides that enable simple and straightforward functional studies of genes involved in neuronal biology and neurodegenerative disorders in a native biological context.

## Introduction

RNA interference (RNAi) is a highly efficient gene-silencing mechanism in which a small interfering RNA (siRNA) binds a target mRNA, guiding mRNA cleavage via an RNA-induced silencing complex (RISC).^1,2^ This biological phenomenon is widely used as a genetic tool in biomedical research. Advances in RNA chemistry have expanded siRNA applications toward therapeutic development, with robust efficacy seen in phase 2 clinical trials for liver diseases (*e.g.*, transthyretin amyloidosis).^3,4,5^

Despite its prevalence in biomedical research, the use of RNAi in neurodegenerative research has been limited.^6^ There is a significant unmet need for simple, effective, and nontoxic siRNA delivery methods to modulate gene expression in primary neurons and brain. A range of approaches has been evaluated,^7^ including AAV viruses,^8,9^ peptide conjugates,^10^ oligonucleotide formulations,^11^ infusion of naked or slightly modified siRNAs,^12,13^ ultrasound,^14^ and convection-enhanced based delivery.^15^ None of these approaches has received wide acceptance due to toxicity, a requirement for extensive repetitive dosing, and/or limited spatial distribution. Lipofection and electroporation of siRNAs are challenging in primary neurons due to low transfection efficiencies and their extreme sensitivity to external manipulation.^16^ Delivery of siRNA precursors (Lentiviruses and AAV) has been used successfully, but viral transduction cannot readily be turned off and requires extensive formulation and experimental optimization to achieve reproducible, nontoxic silencing in neuronal cells.^17,18,19,20,21,22^

**Figure 1:**
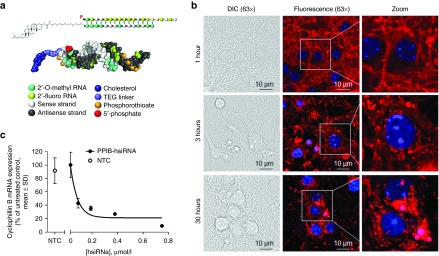
**hsiRNAs are efficiently internalized by primary cortical neurons**. (**a**) Schematic structure of hsiRNAs. A double-stranded oligonucleotide with single-stranded, phosphorothioated tale. 2′-O-methyl and 2′-fluoro modifications, conjugated to teg-chol. (**b**) Fluorescent images of primary cortical neurons incubated with 0.5 µmol/l Cy3-PPIB hsiRNA (red). Nuclei counterstained with Hoechst dye (blue), imaged on Zeiss confocal microscope, ×63. Bar = 10 µm. Images are representative, results confirmed in five separate experiments. (**c**) Primary cortical neurons incubated for 72 hours with hsiRNA targeting *Ppib* at concentrations shown. Level of *Ppib* mRNA was measured using QuantiGene (Affymetrix) normalized to housekeeping gene, *Htt*, presented as percent of untreated control (*n* = 3 wells, mean ± SD). NTC, nontargeting control (0.75 µmol/l). Graph is representative, results confirmed in three separate experiments.

In this study, we describe the delivery, distribution, and silencing capacity of hydrophobically modified siRNAs (hsiRNAs) in primary neurons and in mouse brain. hsiRNAs are siRNA-antisense hybrids containing numerous chemical modifications (see **Figure 1** and **Supplementary Table S1** for exact chemical composition of compounds used) designed to promote biodistribution and stability while minimizing immunogenicity. As a model for our studies, we silenced the huntingtin (*Htt*) gene, the causative gene in Huntington's disease (HD). HD is an autosomal-dominant neurodegenerative disorder caused by a toxic expansion in the CAG repeat region of the huntingtin gene leading to a variety of molecular and cellular consequences. Tetrabenazine, the only FDA-approved therapy for HD, seeks to alleviate disease symptoms but does not treat the actual problem: the gain of toxic function caused by mutant *Htt*. Recent studies suggest that transient neuronal knockdown of *Htt* mRNA can reverse disease progression without compromising normal cellular function *in vivo*.^23^ At present, RNA interference via siRNA or antisense oligonucleotide is one of the most promising therapeutic approaches for transient *Htt* mRNA silencing.

We performed a screen of hsiRNAs targeting *Htt* mRNA and identified multiple functional compounds. We showed that primary neurons internalize hsiRNA added directly to the culture medium, with membrane saturation occurring by 1 hour. Direct uptake in neurons induces potent and long-lasting silencing of *Htt* mRNA for up to 3 weeks *in vitro* without major detectable effects on neuronal viability. Additionally, a single injection of unformulated (without cationic lipid or AAV formulation) *Htt* hsiRNA into mouse brain silences *Htt* mRNA with minimal neuronal toxicity.

Efficient gene silencing in primary neurons and *in vivo* upon direct administration of unformulated hsiRNA represents a significant technical advance in the application of RNAi to neuroscience research, enabling technically achievable genetic manipulation in a native, biological context.

## Results

### hsiRNAs are efficiently internalized by primary neurons

hsiRNA is an asymmetric compound composed of a 15-nucleotide modified RNA duplex with a single-stranded 3′ extension on the guide strand (**Figure 1a** and **Supplementary Table S1**).^24,25^ Pyrimidines in the hsiRNA are modified with 2′-O-methyl (passenger strand) or 2′-fluoro (guide strand) to promote stability, and the 3′ end of the passenger strand is conjugated to a hydrophobic teg-Chol (tetraethylene glycol cholesterol) to promote membrane binding and association.^26^ The single-stranded tail contains hydrophobic phosphorothioate linkages and promotes cellular uptake by a mechanism similar to that of antisense oligonucleotides.^27^ The presence of phosphorothioates, ribose modifications, and a cholesterol conjugate contribute to overall hydrophobicity and are essential for compound stabilization and efficient cellular internalization.

Previous studies have shown that hydrophobically modified siRNAs bind to a wide range of cells and is readily internalized without the requirement for a transfection reagent.^26,28,29^ Here, we evaluated whether asymmetric hydrophobically modified siRNAs are efficiently internalized by primary neurons. We found that, when added to the culture medium, Cy3-labeled hsiRNAs rapidly associated with primary cortical neurons (**Figure 1b**). These Cy3-labeled hsiRNAs were observed in every cell in the culture, demonstrating efficient and uniform uptake. Initially, hsiRNAs mainly associate with neurites and, over time, accumulate in the cell bodies. Treatment of primary neurons with a previously identified hsiRNA targeting *Ppib*^26,28^ (encodes cyclophilin B) reduced target mRNA levels by 90%, further supporting that the observed compound internalization results in potent gene silencing (**Figure 1c**).

### Identification of hsiRNAs that silence huntingtin mRNA

**Figure 2:**
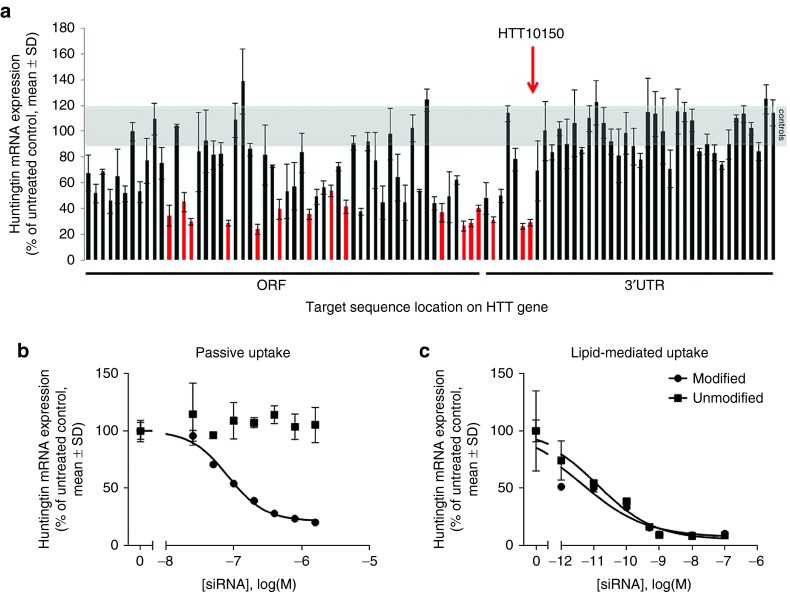
**Systematic screen identifies functional hsiRNAs targeting huntingtin mRNA**. (**a**) Huntingtin mRNA levels in HeLa cells treated for 72 hours with 94 hsiRNAs (1.5 µmol/l) were quantified using QuantiGene and normalized to the housekeeping gene *Ppib*. Data are presented as percent of untreated control (*n* = 3 wells, mean ± SD). Gray area represents range of huntingtin mRNA levels encompassing untreated and nontargeting hsiRNA controls. Red bars indicate compounds selected for further analysis. Compound sequence, chemical composition, and level of silencing are shown in **Supplementary Table S1**. Graph is representative, results confirmed in two separate experiments. (**b**,**c**) Dose–response analysis of huntingtin mRNA levels in HeLa cells treated with HTT10150 hsiRNA (circles) or unmodified siRNA (squares) added to culture medium in the (**b**) absence (modified HTT10150 IC50 = 82.2 nmol/l) or (**c**) presence (modified HTT10150 IC50 = 0.004 nmol/l, unmodified HTT10150 IC50 = 0.013 nmol/l), of cationic lipids for 72 hours. Huntingtin mRNA was measured as described in **a** (*n* = 3 wells, mean ± SD). IC_50_ values were calculated as described in Materials and Methods and are presented in **Supplementary Table S1**. Graph is representative, results for modified siRNA confirmed in three separate experiments (in both absence and presence of cationic lipids), results for unmodified siRNA confirmed in two separate experiments (in both absence and presence of cationic lipids).

Robust uptake and efficacy observed with hsiRNAs in primary cortical neurons encouraged us to identify functional compounds that target *Htt* mRNA, the single gene responsible for the development of Huntington's disease. The hsiRNA's extensive chemical scaffold^26,28^ is essential for stability, minimization of innate immune response,^30,31^ and cellular internalization but imposes significant restrictions on sequence space by potentially interfering with the compound's RISC-entering ability. To maximize the likelihood of identifying functional *Htt* hsiRNAs and to evaluate the hit rate for this type of chemistry, we designed (using conventional criteria described in Materials and Methods) and synthesized hsiRNAs targeting 94 sites across the human *Htt* mRNA (**Supplementary Table S1**). The panel of hsiRNAs was initially screened for efficacy in HeLa cells by adding hsiRNA directly to the culture medium (without lipofection or electroporation) to a final concentration of 1.5 µmol/l and evaluating impact on levels of *Htt* and housekeeping (*Ppib*) gene mRNA expression using the QuantiGene (Affymetrix, Santa Clara, CA) assay. At this concentration, 24 hsiRNAs reduced *Htt* mRNA levels to less than 50% of control levels, including 7 hsiRNAs that reduced *Htt* mRNA levels below 30% of control (**Figure 2a**). Unlike unmodified siRNA libraries, creating a library with extensive 2'-O-methyl and 2'-fluoro modifications introduces additional constraints on sequence selection. As a result, hit rates for modified siRNA screens are lower than that seen for conventional unmodified siRNA.^32,33,34,35^ Functional hsiRNAs targeted sites distributed throughout the mRNA, except the distal end of the 3′ UTR, which later was shown to be part of the alternative *Htt* gene isoform^36^ not expressed in HeLa cells (data not shown). Discounting the ~32 hsiRNAs targeting long 3′ UTR sites absent from the *Htt* isoform in HeLa cells, almost 40% of hsiRNAs showed some level of activity at 1.5 µmol/l, demonstrating that the evaluated chemical scaffold is well tolerated by the RNAi machinery and a functional compound can be easily identified against a wide range of targets.

Half-maximal inhibitory concentrations (IC_50_) for passive uptake of hsiRNAs ranged from 82 to 766 nmol/l (**Supplementary Table S1 and Figure S1**). In lipid-mediated delivery, eight of the most active hsiRNAs had IC_50_ values ranging from 4 to 91 pmol/l (**Supplementary Table S1**). The best clinically active siRNAs are usually characterized by IC_50_ values in the low pmol/l range.^37^ An ability to identify highly potent compounds with low picomolar IC_50_ values suggests that the hsiRNA chemical scaffold does not interfere with siRNA biological activity in selected compounds. The most potent hsiRNA targeting position, 10150 (HTT10150), and an unmodified conventional siRNA version of HTT10150 showed similar IC_50_ values in lipid-mediated delivery (4 and 13 pmol/l respectively, **Figure 2c**), further confirming that the hsiRNA chemical scaffold does not interfere with RISC loading or function. Only the fully modified hsiRNA, and not the unmodified version, silenced *Htt* mRNA by passive uptake (**Figure 2b**). Thus, the chemical scaffold described here does not interfere with RISC assembly and is sufficient to support unformulated compound uptake and efficacy. HTT10150 was used for subsequent studies.

### Potent and specific silencing with unformulated hsiRNAs in primary neurons

**Figure 3:**
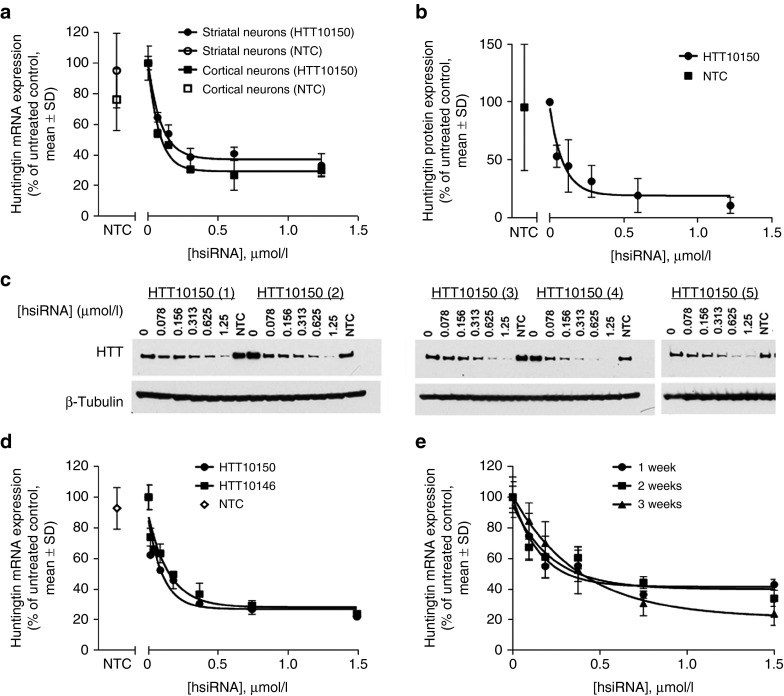
**HTT10150 shows dose-dependent silencing of huntingtin by passive uptake in primary neurons**. (**a**) Huntingtin mRNA levels in primary striatal (black) or cortical (gray) neurons 1 week after treatment with the indicated concentrations of HTT10150. Huntingtin mRNA levels were normalized to *Ppib* mRNA. Data are expressed as percent of untreated control (*n* = 3 wells, mean ± SD). NTC, nontargeting control (1.25 µmol/l). (**b**) Huntingtin protein levels in primary neurons 1 week after treatment with the indicated concentrations of HTT10150. Huntingtin and β-tubulin proteins were quantified by densitometry of western blots, and huntingtin protein levels were normalized to β-tubulin. Data are expressed relative to the level of huntingtin protein in untreated control cells. (*n* = 5 neuronal preparations from separate pups, mean ± SD). NTC, nontargeting control (1.25 µmol/l). Graph of silencing in primary cortical neurons after 1 week is representative, results confirmed in five separate experiments. (**c**) Original western blots from graph in **b**. Primary cortical neurons were cultured from five individual pups (#1–5) and incubated with HTT10150 at concentrations shown for 1 week. Huntingtin protein levels were detected by western blot using antibody AB1 (Huntingtin 1–17). NTC, nontargeting control. (**d**) Primary neurons were incubated with HTT10150 at concentrations shown, for 1, 2, and 3 weeks. Level of huntingtin mRNA was measured using QuantiGene (Affymetrix) normalized to housekeeping gene, *Ppib* (cyclophillin B), and presented as percent of untreated control (*n* = 3 wells, mean ± SD). NTC, nontargeting control (1.5 µmol/l). Graph of silencing in primary cortical neurons after 1 week is representative, results confirmed in five separate experiments. (**e**) Primary cortical neurons were incubated with two different *Htt* hsiRNA sequences HTT10150 and HTT10146 at concentrations shown for 72 hours. Level of huntingtin mRNA was measured using QuantiGene (Affymetrix) normalized to housekeeping gene, *Ppib* (cyclophillin B), and presented as percent of untreated control (*n* = 3 wells, mean ± SD). NTC, nontargeting control (1.5 µmol/l). Graph of HTT10150 silencing in primary cortical neurons after 72 hours is representative, results confirmed in seven separate experiments.

HTT10150 induced a concentration-dependent silencing at 72 hours and 1 week after unformulated addition to either primary cortical or primary striatal neurons isolated from FVB/NJ mice (**Figure 3a**). At 1.25 µmol/l, HTT10150 induced maximal silencing, reducing both *Htt* mRNA levels and HTT protein levels by as much as 70 and 85%, respectively (**Figure 3a**–**c** for original westerns). HTT10150 hsiRNA did not affect the expression levels of housekeeping controls (*Ppib* and *Tubb1*) or the overall viability of primary neuronal cells, as measured by the alamarBlue assay, up to a 2 µmol/l concentration (**Supplementary Figure S2**). Similar results were obtained with another hsiRNA targeting *Htt* mRNA (**Figure 3c**), supporting that the observed phenomena is not unique to HTT10150. These experiments, in conjugation with the results seen from targeting *Ppib* (**Figure 1b**), indicate that a diversity of genes and target sequences can be silenced by hsiRNAs in primary neurons simply upon direct addition of compounds into cellular media.

Since loaded RISC has a typical half-life of weeks,^38^ silencing is expected to be long lasting in nondividing cells. To evaluate duration of silencing after a single HTT10150 treatment of primary cortical neurons, *Htt* mRNA levels were measured at 1-, 2-, and 3-week intervals (**Figure 3d**). A single treatment with hsiRNA induced *Htt* silencing that persisted for at least 3 weeks, the longest time that primary cortical neurons can be maintained in culture. Together, these data demonstrate that hsiRNAs are a simple and straightforward approach for potent, specific, nontoxic, and long-term modulation of gene expression in primary neurons *in vitro*.

### hsiRNA distribution *in vivo* in mouse brain after intrastriatal injection

**Figure 4:**
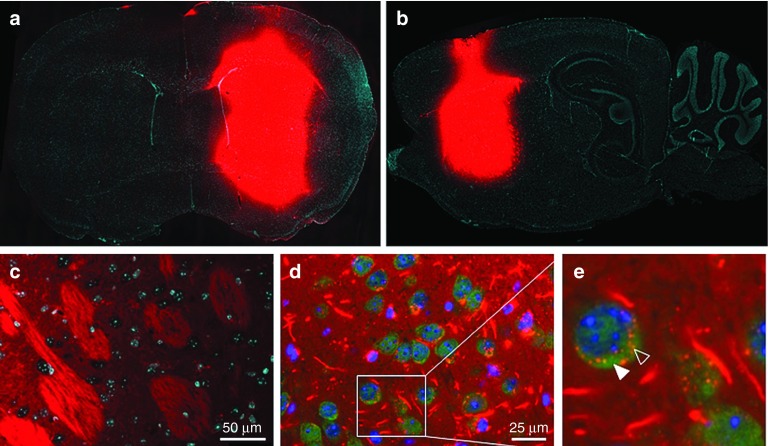
**A single intrastriatal injection of HTT10150 is localized to neurons and fiber tracts ipsilateral to the injection site after 24 hours**. Twenty-five micrograms of Cy3-HTT10150 (red) was unilaterally injected into the striatum of WT (FVB/NJ) mice. Brains were collected after 24 hours, paraffin embedded, and sectioned. (**a**) Tiled image of coronal brain section (×16). Majority of HTT10150 is localized at site of injection with sharp gradient of diffusion. (**b**) Tiled image of sagittal brain section (×16), injected side. (**c**) Image of coronal brain section (×40), injected side. (**d**) Image of coronal brain section (×60), injected side, with NeuN-stained neurons. (**e**) NeuN-stained neurons from injected side (×60) zoomed in. Solid arrow, NeuN staining. Open arrow, Cy3-HTT10150 punctae in perinuclear space. Images are representative, results confirmed in two separate experiments.

Having shown that hsiRNAs effectively silence their targets in primary neurons *in vitro*, we sought to evaluate the ability of HTT10150 to silence *Htt* mRNA in the mouse brain *in vivo*. The distribution of HTT10150 was evaluated in perfused brain sections prepared 24 hours after intrastriatal injection with 12.5 µg Cy3-labeled hsiRNA in artificial cerebral spinal fluid (ACSF). We observed a steep gradient of fluorescence emanating from the injection site and covering most of the ipsilateral striatum (**Figure 4a**,**b**), while no fluorescence was visually detectable in the contralateral side of the brain. In high magnification images of the ipsilateral side, hsiRNAs appeared preferentially associated with the tissue matrix and fiber tracts. In addition, efficient internalization was observed in a majority of cell bodies (**Figure 4c**,**d**). Consistent with *in vitro* studies, we observed Cy3-labeled hsiRNA in neuronal processes and as punctae in the perinuclear space of multiple cell types, including NeuN-positive neurons^39,40^ (**Figure 4d**,**e**). In summary, a single intrastriatal injection delivers hsiRNA to neurons in the striatum of the injected side.

### hsiRNA effectively silences Htt in vivo with minimal cytotoxicity or immune activation

**Figure 5:**
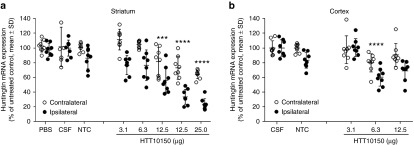
**HTT10150 effectively silences huntingtin mRNA ipsilateral to the site of injection**. HTT10150 was unilaterally injected into the striatum of WT (FVB/NJ) mice (2 µl). Mice were sacrificed at 5 days. Brains were sliced into 300-μm sections and six 2-mm punch biopsies of the (**a**) striatum and (**b**) cortex were collected from both ipsilateral and contralateral sides. Level of huntingtin mRNA was measured using QuantiGene (Affymetrix) normalized to housekeeping gene, *Ppib* (cyclophillin B), and presented as percent of untreated control (*n* = 8 mice, mean ± SD, three biopsies per region). *P* values are all calculated for each dose group relative to NTC by Two-way repeated-measures analysis of variance: 25 µg striatum, *P* < 0.0001; 12.5 µg striatum, *P* < 0.0001, *P* = 0.0002; 6.3 µg cortex, *P* = 0.0009. NTC, nontargeting control.

To measure HTT10150 efficacy *in vivo*, we performed dose-response studies in wild type FVB/NJ mice injected intrastriatally with 3.1, 6.3, 12.5, or 25 µg of HTT10150. As controls, we injected mice with a non-targeting control hsiRNA (NTC), ACSF, or PBS. In punch biopsies taken from the ipsilateral and contralateral striatum, HTT10150 reduced *Htt* mRNA levels in a dose-dependent manner (**Figure 5a**).

This experiment was repeated several times with similar results. The *Htt* mRNA is significantly reduced in the ipsilateral side of striatum in all experiments. We observed robust dose-dependent silencing with up to 77% (one-way analysis of variance, *P* < 0.0001) reduction in *Htt* mRNA expression levels at the highest dose. Interestingly we observe statistically significant, but less pronounced silencing in the contralateral striatum and the cortex. The silencing reaches statistical significance with both one-way and two-way analysis of variance (values for two-way analysis of variance are presented in **Figure 5**). While some level of fluorescence is detectable in these brain regions with high laser intensity, it is very close to the tissue auto-fluorescence and thus is not reported here. We will be investigating this phenomenon further, but it is clear that the level of silencing is at least correlative to the sharp gradient of diffusion from the injection site.

Finally, *Htt* mRNA silencing is observed with HTT10150 but not with NTC or ACSF (**Figure 5**). In addition, the HTT10150 does not affect expression of several housekeeping genes (PPIB, HPRT). In combination, this is indicative of *Htt* mRNA silencing being caused by HTT10150 hsiRNA and not by off-target effects.

**Figure 6:**
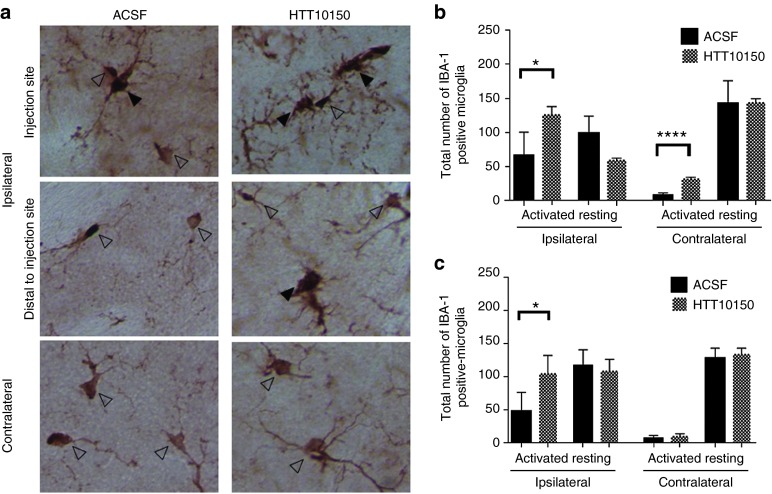
**HTT10150 shows a twofold increase in microglial activation at the site of injection**. HTT10150 was unilaterally injected into the striatum of WT (FVB/NJ) mice. Brains were collected after (**b**) 6 hours and (**a**, **c**) 5 days fixed, sectioned, and stained with antibodies against IBA-1. (**a**) Representative images of activated (black arrow) and resting (open arrow) after injection of 12.5 µg HTT10150 and ACSF 5 days post-injection, ×40 magnification. (**b**) Quantification of activated and resting microglia 6 hours post-injection of ACSF (*n* = 6 mice, mean ± SD) and 12.5 µg HTT10150 (*n* = 3 mice, mean ± SD). *P* values calculated by unpaired *t*-test, *t* = 9.996, *df* = 7: ACSF versus HTT10150 activated microglia ipsilateral striatum, *P* = 0.0239. ACSF versus HTT10150 activated microglia contralateral striatum, *P* < 0.0001. (**c**) Quantification of activated and resting microglia 5 days postinjection of ACSF (*n* = 4 mice, mean ± SD) and 12.5 µg HTT10150 (*n* = 3 mice, mean ± SD). Images are representative, results confirmed in separate images of all injected brains. *P* values calculated by unpaired *t*-test, *t* = 2.700, *df* = 5: ACSF versus HTT10150 activated microglia ipsilateral striatum *P* = 0.0428.

Nucleic acids, including siRNAs, are potent stimulators of the innate immune response,^41^ but extensive chemical modifications, like 2′-O-methyl, are expected to suppress the immunostimulatory effects of siRNAs *in vitro* and *in vivo*.^42^ To assess innate immune response activation by hsiRNAs *in vivo*, we quantified IBA-1-positive microglial cells in brain sections from mice injected with 12.5 µg HT10150 or artificial CSF. IBA-1 is specific to microglial cells and is upregulated following injury to the brain, allowing us to distinguish between resting and activated microglia.^43,44,45^ In the case of a major innate immune response, an increase of 200–300% in total microglia is customary.^46^ Total microglia counts showed only a 25% increase in the ipsilateral striatum at 5 days post-injection indicating a lack of any major inflammatory response (**Supplementary Figure S3**). Thus, the observed activation is relatively minor but reaches statistical significance, indicating some level of response. Levels of innate immune response might be more pronounced immediately following compound administration. To assess the level of stimulation in more detail, we separately evaluated the number of activated and resting microglia at both 6 hours and 5 days post-injection. At 6 hours post-injection, we observed a significant increase in the number of activated microglia in the injected side of the brain with both ACSF and HTT10150. The injection event itself causes trauma and induces a major increase in activated microglia (ninefold) compared to the contralateral side of the brain (**Figure 6b**).^12,47^ In the presence of HTT10150, the number of activated microglia was additionally increased twofold compared to ACSF, indicating enhancement of trauma-related microglia activation in the presence of oligonucleotide, although the relative contribution of the oligonucleotide to the trauma-related induction is minor. HTT10150-treated mice also showed some elevation of activated microglia in the contralateral striatum 6 hours post-injection (**Figure 6b**); however, after 5 days, all changes in number of microglia in the contralateral side of the brain disappeared (**Figure 6a**,**c** for representative images), suggesting that HTT10150-dependent activation of microglia in the contralateral striatum is transient.

**Figure 7:**
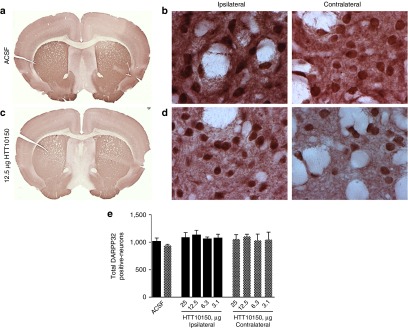
**HTT10150 shows no toxicity in DARPP-32-positive neurons around the site of injection**. HTT10150 was unilaterally injected into the striatum of WT (FVB/NJ) mice. Brains were collected after 5 days, fixed, sectioned, and stained with antibodies against DARPP-32 (**a–d**). Representative image of striatum after injection of (**a**,**b**) ACSF, full brain scan and ×60 magnification or (**c,d**) 12.5 µg HTT10150, full brain scan and ×60 magnification. (**a**) Quantification of DARPP-32–positive neurons (*n* = 3 mice, mean ± SD). Images are representative results confirmed in separate images of all injected brains.

Despite the mild immune stimulation in the brains of animals injected with HTT10150, we did not observe any overall significant reduction of DARPP-32, an established marker for striatal neuron viability^48^ (**Figure 7**). The only observed effect was at a small area directly around the injection site in animals treated with 25 µg HTT10150 (**Supplementary Figure S4**). Taken together, our data show that a single intrastriatal injection of hsiRNA induces potent gene silencing with a mild immune response and minimal neuronal toxicity *in vivo*.

## Discussion

Simple, effective, and nontoxic delivery of synthetic oligonucleotides to primary neurons and brain tissue represents a challenge to the use of RNAi as a research tool and therapeutic for neurodegenerative diseases like HD.^7^ We have shown that hsiRNAs elicit potent silencing in primary neurons in culture, without effect on housekeeping gene expression, and with minimal toxicity at effective doses Additionally, a non-targeting control hsiRNA did not silence any of the mRNAs tested (*Htt*, *Ppib*, *Hprt*), suggesting that these compounds are both sequence specific and on-target. Interestingly, the level of silencing is more pronounced on the protein level (>90%) compared to the mRNA level (>70%). The mRNA plateau effect is reproducible and is specific to *Htt* mRNA, as housekeeping genes like *Ppib* can be silenced by 90%. One potential explanation is that some fraction of huntingtin mRNA is translationally inactive and poorly accessible by RNAi machinery. We are continuing to investigate this phenomenon. Silencing in primary neurons persists for multiple weeks after a single administration, consistent with the expected half-life of active RISC.^49^ Moreover, efficient intracellular delivery does not require the use of lipids or viral packaging.

Currently, the most impressive *in vivo* modulation of *Htt* mRNA expression is demonstrated with 2′-O-methoxyethyl GapmeR antisense oligonucleotides. A single injection of 50 µg of antisense oligonucleotides or infusion of around 500 µg results in potent and specific *Htt* mRNA silencing and marked improvement in multiple phenotypic endpoints.^23,50,51,52^ However, 2′-O-methoxyethyl GapmeR antisense oligonucleotides are not readily commercially available making them inaccessible for the majority of academic labs.

Here, we show *Htt* mRNA silencing in the ipsilateral striatum and cortex, two brain areas significantly affected in HD disease progression, with a single intrastriatal injection. As a considerably reduced level of silencing was observed on the contralateral side of the brain, bilateral injections might be necessary to promote equal gene silencing in both hemispheres.

The limited distribution profile observed *in vivo* restricts immediate adoption of this technology for use in larger brains and eventually as a therapeutic for neurodegenerative disease. Tissue distribution can be improved by tailoring the chemical scaffold (*e.g.*, number and type of sugar modifications, position of phosphorothioate linkages) or by changing the conjugation moiety to promote receptor-mediated cellular internalization. Formulation of hsiRNA in exosomes, exosome-like liposomes, or shielding the compounds with polyethylene glycol may also provide an alternative strategy to improve tissue distribution.^53,54^

Here, we describe a class of self-delivering therapeutic oligonucleotides capable of targeted, nontoxic, and efficient *Htt* gene silencing in primary neurons and *in vivo*. This chemical scaffold can be specifically adapted to many different targets to facilitate the study of neuronal gene function *in vitro* and *in vivo*. The development of an accessible strategy for genetic manipulation in the context of a native, biological environment represents a technical advance for the study of neuronal biology and neurodegenerative disease.

## Materials and methods

*hsiRNA design.* We designed and synthesized a panel of 94 hsiRNA compounds (**Supplementary Table S1**) targeting the human huntingtin gene. These sequences span the gene and were selected to comply with standard siRNA design parameters^24^ including assessment of GC content, specificity and low seed compliment frequency,^55^ elimination of sequences containing miRNA seeds, and examination of thermodynamic bias.^56,57^

*Oligonucleotide synthesis, deprotection, and purification.* Oligonucleotides were synthesized using standard phosphoramidite, solid-phase synthesis conditions on a 0.2–1 µmole scale using a MerMade 12 (BioAutomation, Irving, TX) and Expedite DNA/RNA synthesizer. Oligonucleotides with unmodified 3′ ends were synthesized on controlled pore glass (CPG) functionalized with long-chain alkyl amine and a Unylinker terminus (Chemgenes, Wilmington, MA). Oligonucleotides with 3′-cholesterol modifications were synthesized on modified solid support (Chemgenes). Phosphoramidite solutions were prepared at 0.15 mol/l in acetonitrile for 2′-TBDMS, 2′-O-methyl (Chemgenes), and Cy3 modifications or 0.13 mol/l for 2′-fluoro (BioAutomation) modifications. Phosphoramidites were activated in 0.25 mol/l 4,5-dicyanoimidazole in acetonitrile. Detritylation was performed in 3% dichloroacetic acid in dichloromethane for 80 seconds. Capping was performed in 16% *N*-methylimidazole in tetrahydrofuran and acetic anhydride:pyridine:tetrahydrofuran, (1:2:2, v/v/v) for 15 seconds. Oxidation was performed using 0.1 mol/l iodine in pyridine:water:tetrahydrofuran (1:2:10, v/v/v).

The CPG was removed from the solid-phase column and placed in a polypropylene screw cap vial. Dimethylsulfoxide (100 µl) and 40% methylamine (250 µl) are added directly to the CPG and shaken gently at 65 °C for exactly 16 minutes. The vial was cooled on dry ice before the cap was removed. The supernatant was transferred to another polypropylene screw cap vial, and the CPG was rinsed with two 150 µl portions of dimethylsulfoxide, which were combined with original supernatant. Oligonucleotides without 2′-TBDMS-protecting groups were lyophilized. Oligonucleotides with 2′-TBDMS-protecting groups were desilylated by adding 375 µl triethylamine trihydrofluoride (~1.5 volumes relative to 40% methylamine) and incubated for exactly 16 minutes at 65 °C with gentle shaking. Samples were quenched by transferring to a 15 ml conical tube containing 2 ml of 2 mol/l triethylammonium acetate buffer (pH 7.0). The sample was stored at −80 °C until high-performance liquid chromatography purification.

Oligonucleotides were purified by reverse-phase high-performance liquid chromatography on a Hamilton PRP-C18 column (21 × 150 mm) using an Agilent Prostar 325 high-performance liquid chromatography (Agilent, Santa Clara, CA). Buffer A 0.05 mol/l tetraethylammonium acetate with 5% acetonitrile, Buffer B 100% acetonitrile, with a gradient of 0% B to 35% B over 15 minutes at 30 ml/minutes. Purified oligonucleotides were lyophilized to dryness, reconstituted in water, and passed over a Hi-Trap cation exchange column to exchange the tetraethylammonium counter-ion with sodium.

*Cell culture.* HeLa cells (ATCC, Manassas, VA; #CCL-2) were maintained in Dulbecco's Modified Eagle's Medium (Cellgro, Corning, NY; #10-013CV) supplemented with 10% fetal bovine serum (FBS; Gibco, Carlsbad, CA; #26140) and 100 U/ml penicillin/streptomycin (Invitrogen, Carlsbad, CA; #15140) and grown at 37 °C and 5% CO_2_. Cells were split every 2 to 5 days and discarded after 15 passages.

*Preparation of primary neurons.* Primary cortical neurons were obtained from FVB/NJ mouse embryos at embryonic day 15.5. Pregnant FVB/NJ females were anesthetized by intraperitoneal injection of 250 mg Avertin (Sigma, St Louis, MO; #T48402) per kg weight, followed by cervical dislocation. Embryos were removed and transferred into a Petri dish with ice-cold Dulbecco's Modified Eagle's Medium/F12 medium (Invitrogen; #11320). Brains were removed, and meninges carefully detached. Cortices were isolated and transferred into a 1.5-ml tube with prewarmed papain solution for 25 minutes at 37 °C, 5% CO_2_, to dissolve tissue. Papain solution was prepared by suspending DNase I (Worthington, Lakewood, NJ; #54M15168) in 0.5 ml Hibernate E medium (Brainbits, Springfield, IL; #HE), and transferring 0.25 ml DNase I solution to papain (Worthington, Lakewood, NJ; #54N15251) dissolved in 2 ml Hibernate E medium and 1 ml Earle's balanced salt solution (Worthington; #LK003188). After the 25-minute incubation, papain solution was replaced with 1 ml NbActiv4 medium (Brainbits; #Nb4-500) supplemented with 2.5% FBS. Cortices were dissociated by repeated pipetting with a fire-polished, glass, Pasteur pipette. Cortical neurons were counted and plated at 1 × 10^6^ cells per ml.

For live-cell imaging, culture plates were precoated with poly-l-lysine (Sigma; #P4707), and 2 × 10^5^ cells were added to the glass center of each dish. For silencing assays, neurons were plated on 96-well plates precoated with poly-l-lysine (BD BIOCOAT, Corning, NY; #356515) at 1 × 10^5^ cells per well. After overnight incubation at 37 °C, 5% CO_2_, an equal volume of NbActiv4 supplemented with anti-mitotics, 0.484 µl/ml of UTP Na_3_ (Sigma; #U6625), and 0.2402 µl/ml of FdUMP (Sigma; #F3503), was added to neuronal cultures to prevent growth of nonneuronal cells. Half of the media volume was replaced every 48 hours until the neurons were treated with siRNA. Once the cells were treated, media was not removed, only added. All subsequent media additions contained antimitotics.

*Direct delivery (passive uptake) of oligonucleotides.* Cells were plated in Dulbecco's Modified Eagle's Medium containing 6% FBS at 10,000 cells per well in 96-well tissue culture plates. hsiRNA was diluted to twice the final concentration in OptiMEM (Gibco; #31985-088), and 50 μl diluted hsiRNA was added to 50 μl of cells, resulting in 3% FBS final. Cells were incubated for 72 hours at 37 °C and 5% CO_2_. Based on previous experience, we know that 1.5 µmol/l active hsiRNA supports efficient silencing without toxicity. The primary screen for active *Htt* siRNAs, therefore, was performed at 1.5 µmol/l compound, which also served as the maximal dose for *in vitro* dose–response assays.

*hsiRNA lipid-mediated delivery.* Cells were plated in Dulbecco's Modified Eagle's Medium with 6% FBS at 10,000 cells per well in 96-well tissue culture–treated plates. hsiRNA was diluted to four times the final concentration in OptiMEM, and Lipofectamine RNAiMAX Transfection Reagent (Invitrogen; #13778150) was diluted to four times the final concentration (final = 0.3 µl/25 µl/well). RNAiMAX and hsiRNA solutions were mixed 1:1, and 50 µl of the transfection mixture was added to 50 µl of cells resulting in 3% FBS final. Cells were incubated for 72 hours at 37 °C and 5% CO_2_.

*mRNA quantification in cells and tissue punches.* mRNA was quantified using the QuantiGene 2.0 Assay (Affymetrix; #QS0011). Cells were lysed in 250 μl diluted lysis mixture composed of 1 part lysis mixture (Affymetrix; #13228), 2 parts H_2_O, and 0.167 μg/μl proteinase K (Affymetrix; #QS0103) for 30 minutes at 55 °C. Cell lysates were mixed thoroughly, and 40 μl (~8,000 cells) of each lysate was added per well to a capture plate with 40 μl diluted lysis mixture without proteinase K. Probe sets were diluted as specified in the Affymetrix protocol. For HeLa cells, 20 μl human *HTT* or *PPIB* probe set (Affymetrix; *#*SA-50339, #SA-10003) was added to appropriate wells for a final volume of 100 μl. For primary neurons, 20 μl of mouse *Htt* or *Ppib* probe set (Affymetrix; *#*SB-14150, #SB-10002) was used.

Tissue punches (5 mg) were homogenized in 300 μl of Homogenizing Buffer (Affymetrix; #10642) containing 2 μg/μl proteinase K in 96-well plate format on a QIAGEN TissueLyser II (Qiagen, Valencia, CA; #85300), and 40 μl of each lysate was added to the capture plate. Probe sets were diluted as specified in the Affymetrix protocol, and 60 μl of *Htt* or *Ppib* probe set was added to each well of the capture plate for a final volume of 100 μl. Signal was amplified according to the Affymetrix protocol. Luminescence was detected on either a Veritas Luminometer (Promega, Madison, WI; #998–9100) or a Tecan M1000 (Tecan, Morrisville, NC).

*Western blot.* Cell lysates (25 µg) were separated by SDS–PAGE using 3–8% Tris-acetate gels (Life Technologies, Grand Island, NY; #EA03785BOX) and transferred to nitrocellulose using a TransBlot Turbo apparatus (BioRad, Hercules, CA; #1704155). Blots were blocked in 5% nonfat dry milk (BioRad; #1706404) diluted in Tris-buffered saline with 0.1% Tween-20 (TBST) for 1 hour at room temperature then incubated in N-terminal antihuntingtin antibody Ab1^58^ diluted 1:2,000 in blocking solution overnight at 4 °C with agitation. After washing in TBST, blots were incubated in peroxidase-labeled antirabbit IgG (Jackson ImmunoResearch, West Grove, PA; #711035152) diluted in blocking buffer for 1 hour at room temperature, washed in TBST, and proteins were detected using SuperSignal West Pico Chemiluminescent Substrate (Thermo Scientific, Rockford, IL; #34080) and Hyperfilm ECL (GE Healthcare, Buckinghamshire, UK; #28906839). Blots were reprobed with anti-β tubulin antibody (Sigma; #T8328) as a loading control. Films were scanned with a flatbed scanner (Epson Perfection V750 Pro; Epson, Long Beach, CA), and densitometry was performed using NIH ImageJ software to determine total intensity of each band. The huntingtin signal was divided by the tubulin signal to normalize to protein content, and percent of untreated control was determined for each set of samples (*N* = 5).

*Live cell imaging.* To monitor live cell hsiRNA uptake, cells were plated at a density of 2 × 10^5^ cells per 35-mm glass-bottom dish. Cell nuclei were stained with NucBlue (Life Technologies; #R37605) as indicated by the manufacturer. Imaging was performed in phenol red-free NbActiv4 (Brainbits; #Nb4-500). Cells were treated with 0.5 μmol/l Cy3-labeled hsiRNA, and live cell imaging was performed over time. All live cell confocal images were acquired with a Leica DM IRE2 confocal microscope using 63x oil immersion objective (Buffalo Grove, IL), and images were processed using ImageJ (1.47v) software.

*Stereotaxic injections.* FVB/NJ mice (50% male and 50% female for each dose group, 6–8 weeks old) were deeply anesthetized with 1.2% Avertin (Sigma; #T48402) and microinjected by stereotactic placement into the right striatum (coordinates relative to bregma: 1.0 mm anterior, 2.0 mm lateral, and 3.0 mm ventral). For both toxicity (DARPP-32 staining) and efficacy studies, mice were injected with either PBS or artificial CSF (2 μl per striata), 12.5 μg of nontargeting hsiRNA (2 μl of 500 µmol/l stock per striata), 25 μg of HTT10150 hsiRNA (2 μl of 1 mmol/l stock per striata), 12.5 μg of HTT10150 hsiRNA (2 μl of 500 μmol/l stock per striata), 6.3 μg of HTT10150 hsiRNA (2 μl of 250 μmol/l stock per striata), or 3.1 μg of HTT10150 hsiRNA (2 μl of 125 μmol/l stock per striata). For toxicity studies, *n* = 3 mice were injected per group, and for efficacy studies, *n* = 8 mice were injected per group. Mice were euthanized 5 days post-injection, brains were harvested, and three 300-μm coronal sections were prepared. From each section, a 2-mm punch was taken from each side (injected and noninjected) and placed in RNAlater (Ambion, Carlsbad, CA; #AM7020) for 24 hours at 4 °C. Each punch was processed as an individual sample for Quantigene 2.0 assay analysis (Affymetrix) and averaged for a single animal point. All animal procedures were approved by the University of Massachusetts Medical School Institutional Animal Care and Use Committee (protocol number A-2411).

*Immunohistochemistry/immunofluorescence.* Mice were injected intrastriatally with 12.5 µg of Cy3-labeled hsiRNA. After 24 hours, mice were sacrificed and brains were removed, embedded in paraffin, and sliced into 4-μm sections that were mounted on glass slides. Sections were deparaffinized by incubating in Xylene twice for 8 minutes. Sections were rehydrated in serial ethanol dilutions (100%, 95%, and 80%) for 4 minutes each, and then washed twice for 2 minutes with PBS.

For NeuN staining,^39,40^ slides were boiled for 5 minutes in antigen retrieval buffer (10 mmol/l Tris/ 1mmol/l EDTA (pH 9.0)), incubated at room temperature for 20 minutes, and then washed for 5 minutes in PBS. Slides were blocked in 5% normal goat serum in PBS containing 0.05% Tween 20 (PBST) for 1 hour and washed once with PBST for 5 minutes. Slides were incubated with primary antibody (Millipore, Taunton, MA; MAB377, 1:1,000 dilution in PBST) for 1 hour and washed three times with PBST for 5 minutes. Slides were then incubated with secondary antibody (Life Technologies; #A11011, 1:1000 dilution in PBST) for 30 minutes in the dark and washed three times with PBST for 5 minutes each. Slides were then counterstained with 250 ng/ml 4',6-diamidino-2-phenylindole (Molecular Probes, Life Technologies; #D3571) in PBS for 1 minute and washed three times with PBS for 1 minute. Slides were mounted with mounting medium and coverslips and dried overnight before imaging on a Leica DM5500 microscope fitted with a DFC365 FX fluorescence camera.

For toxicity studies, injected brains were harvested after 5 days. For microglial activation studies, brains were harvested after 6 hours or 5 days. Extracted, perfused brains were sliced into 40-µm sections on the Leica 2000T Vibratome (Leica Biosystems, Wetzlar, Germany) in ice-cold PBS. Every sixth section was incubated with DARPP-32 (Abcam, Cambridge, UK; #40801; 1:10,000 in PBS) or IBA-1 (Wako; #019-19741; 1:1,000 in PBS) antibody, for a total of nine sections per brain and eight images per section (four per hemisphere). IBA-1 sections were incubated in blocking solution (5% normal goat serum, 1% bovine serum albumin, 0.2% Triton-X-100, and 0.03% hydrogen peroxide in PBS) for 1 hour, and then washed with PBS. Sections were incubated overnight at 4 °C in primary antibody, anti-Iba1 (polyclonal rabbit anti-mouse/human/rat; dilution: 1:1,000 in blocking solution) (Wako; #019-19741). Sections were then stained with goat antirabbit secondary antibody (1:200 dilution) (Vector Laboratories, Burlingame, CA), followed by a PBS wash, the Vectastain ABC Kit (Vector Laboratories), and another PBS wash. IBA-1 was detected with the Metal Enhanced DAB Substrate Kit (Pierce, Rockford, IL). For DARPP32 staining, sections were washed for 3 minutes in 3% hydrogen peroxide, followed by 20 minutes in 0.2% TritonX-100 and 4 hours in 1.5% normal goat serum in PBS. Sections were incubated overnight at 4 °C in DARPP32 primary antibody (1:10,000 dilution) (Abcam; #40801) made up in 1.5% normal goat serum. Secondary antibody and detection steps were conducted as described for IBA-1 staining. DARPP-32 sections were mounted and visualized by light microscopy with 20× objective on a Nikon Eclipse E600 with a Nikon Digital Sight DSRi1 camera (Nikon, Tokyo, Japan). The number of DARPP-32-positive neurons was quantified manually using the cell counter plug-in on ImageJ for tracking. Activated microglia were quantified by morphology of IBA-1-positive cells^42,43,44,45^ from the same number of sections captured with 40× objective. Counting of both IBA-1- and DARPP-32-positive cells was blinded. Coronal section images were taken with a Coolscan V-ED LS50 35-mm Film Scanner (Nikon, Tokyo, Japan).

*Statistical analysis.* Data were analyzed using GraphPad Prism 6 software (GraphPad Software, Inc., San Diego, CA). Concentration-dependent IC_50_ curves were fitted using a log(inhibitor) versus response–variable slope (four parameters). The lower limit of the curve was set at zero, and the upper limit of the curve was set at 100. For each independent mouse experiment, the level of knockdown at each dose was normalized to the mean of the control group (the noninjected side of the PBS or artificial CSF groups). *In vivo* data were analyzed using a two-way repeated-measures analysis of variance with Tukey's multiple comparisons test for dose and side of brain. Differences in all comparisons were considered significant at *P* values less than 0.05 compared with the NTC- injected group. *P* values reported represent significance of the entire dose group relative to NTC and are not specific to the ipsilateral or contralateral side. For microglial activation, significance was calculated using a parametric, unpaired, two-tailed *t*-test for comparison between dose groups, and paired *t*-test for comparison between ipsilateral and contralateral hemispheres within the same dose group.

**SUPPLEMENTARY MATERIAL** **Figure S1.** Active hsiRNAs silence huntingtin mRNA in a concentration dependent manner in HeLa cells. **Figure S2.** HTT10150 does not affect primary cortical neuron viability. **Figure S3.** HTT10150 causes a slight increase in total resting microglia 5 days post injection. **Figure S4.** HTT10150 shows limited toxicity at the site of injection at the 25 µg dose. **Table S1.** Detailed sequence, chemical modification patterns, and efficacy of hsiRNAs.

## Supplementary Material

Supplementary Figure S1 Active hsiRNAs silence huntingtin mRNA in a concentration dependent manner in HeLa cells.

Supplementary Figure S2 HTT10150 does not affect primary cortical neuron viability.

Supplementary Figure S3 HTT10150 causes a slight increase in total resting microglia 5 days post injection.

Supplementary Figure S4 HTT10150 shows limited toxicity at the site of injection at the 25 µg dose.

Supplementary Table S1 Detailed sequence, chemical modification patterns, and efficacy of hsiRNAs.
