# Supplementary figures and images for: Hydrophobically Modified siRNAs Silence Huntingtin mRNA in Primary Neurons and Mouse Brain
Source: Mol Ther Nucleic Acids. 2015 Dec 1;4(12):e266–. doi: 10.1038/mtna.2015.38 (PMC5014532; doi:10.1038/mtna.2015.38)

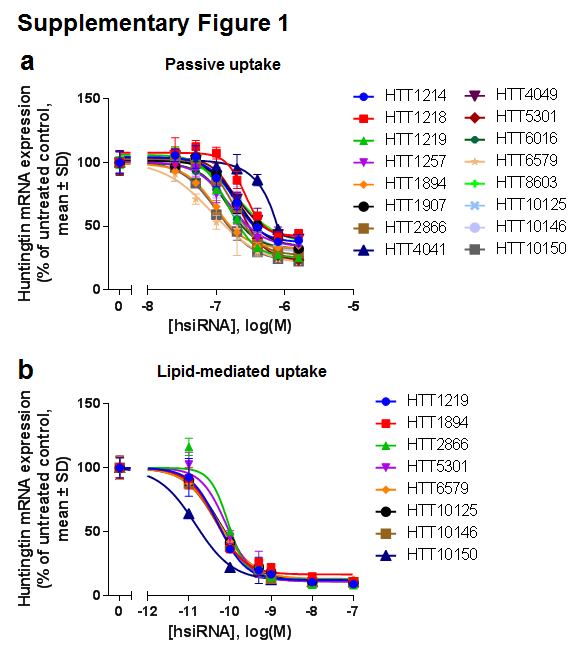

Supplement: Supplementary Figure S1 — Active hsiRNAs silence huntingtin mRNA in a concentration dependent manner in HeLa cells. [file mtna201538x1.tiff]

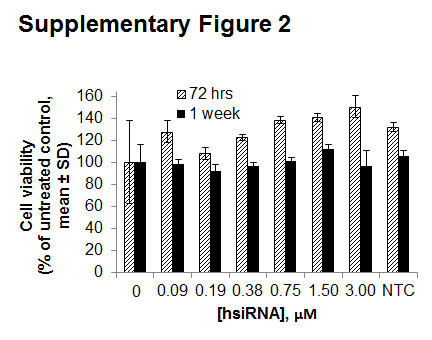

Supplement: Supplementary Figure S2 — HTT10150 does not affect primary cortical neuron viability. [file mtna201538x2.tiff]

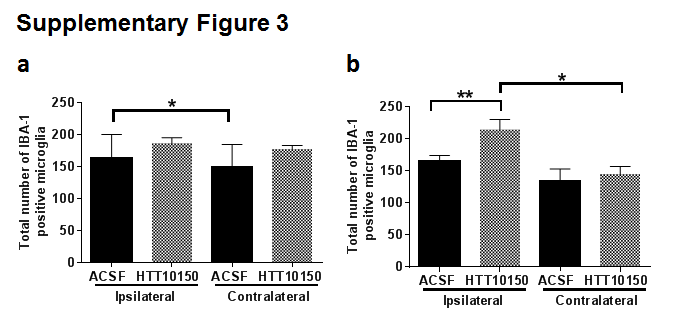

Supplement: Supplementary Figure S3 — HTT10150 causes a slight increase in total resting microglia 5 days post injection. [file mtna201538x3.tiff]

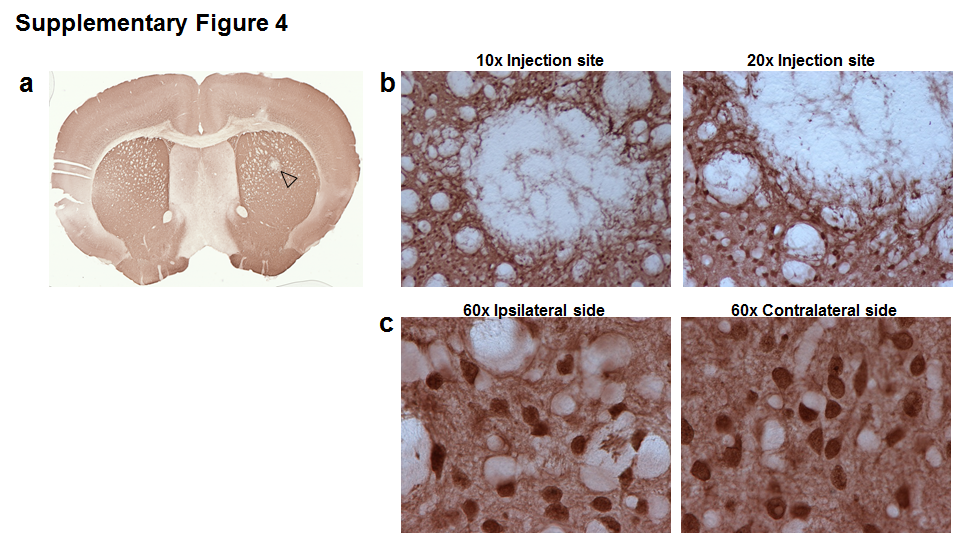

Supplement: Supplementary Figure S4 — HTT10150 shows limited toxicity at the site of injection at the 25 µg dose. [file mtna201538x4.tiff]
