# Supplementary material for: Hydrophobically Modified siRNAs Silence Huntingtin mRNA in Primary Neurons and Mouse Brain
Source: Mol Ther Nucleic Acids. 2015 Dec 1;4(12):e266–. doi: 10.1038/mtna.2015.38 (PMC5014532; doi:10.1038/mtna.2015.38)
Supplement: Supplementary Table S1 — Detailed sequence, chemical modification patterns, and efficacy of hsiRNAs. [file mtna201538x5.docx]

**Table 1** Detailed sequence, chemical modification patterns, and efficacy of hsiRNAs

|  |  |  |  |  |  | **HeLa Cell Activity** | | |
| --- | --- | --- | --- | --- | --- | --- | --- | --- |
|  |  | **Strand Modifications** | |  | | **Primary Screen** | **IC50 (nM)** | |
| **Gene** | **Position** | **Sense Strand** | **Antisense Strand** | **M. musculus** | **M. mulatta** | **Huntingtin mRNA Expression (% control)** | **Passive Uptake** | **Lipid-Mediated Uptake** |
| HTT | 1214 | mG.mG.mU.mU.mU.A.mU.G.A.A.mC.mU.G#mA#mA.tegChol | PmU.fU.fC.A.G.fU.fU.fC.A.fU.A.A.mA.fC#fC#fU#G#G#mA#C | yes | yes | 34.3 | 197.4 | N/A |
| HTT | 1218 | mU.mA.mU.G.A.A.mC.mU.G.A.mC.G.mU#mU#mA.tegChol | PmU.A.A.fC.G.fU.fC.A.G.fU.fU.fC.A.fU#A#A#mA#fC#fC#U |  | yes | 44.8 | 293.2 | N/A |
| HTT | 1219 | mA.mU.G.A.A.mC.mU.G.A.mC.G.mU.mU#mA#mA.tegChol | PmU.fU.A.A.fC.G.fU.fC.A.G.fU.fU.fC.A#fU#A#A#mA#fC#C |  | yes | 29.6 | 163.6 | 0.052 |
| HTT | 1257 | mA.mA.mU.G.mU.mU.G.mU.G.A.mC.mC.G#mG#mA.tegChol | PmU.fC.fC.G.G.fU.fC.A.fC.A.A.fC.A.fU#fU#G#fU#G#G#U |  | yes | 28.5 | 156.7 | N/A |
| HTT | 1894 | mU.mA.G.A.mC.G.G.mU.A.mC.mC.G.A#mC#mA.tegChol | PmU.G.fU.fC.G.G.fU.A.fC.fC.G.fU.fC.fU#A#A#fC#A#fC#A |  | yes | 23.7 | 95.53 | 0.047 |
| HTT | 1907 | mC.mA.A.mC.mC.A.G.mU.A.mU.mU.mU.G#mG#mA.tegChol | PmU.fC.fC.A.A.mA.fU.A.fC.fU.G.G.fU.fU#G#fU#fC#G#G#U |  | yes | 39.3 | 217.9 | N/A |
| HTT | 2866 | mU.mG.mC.mU.mC.A.A.mU.A.A.mU.G.mU#mU#mA.tegChol | PmU.A.A.fC.A.fU.fU.A.fU.fU.G.A.mG.fC#A#fC#fU#fC#G#U |  | yes | 35.3 | 191.7 | 0.091 |
| HTT | 4041 | mU.mC.mC.mU.G.mC.mU.mU.mU.A.G.mU.mC#mG#mA.tegChol | PmU.fC.G.A.fC.fU.A.A.mA.G.fC.A.G.mG#A#fU#fU#fU#fC#A | yes | yes | 53.5 | 765.7 | N/A |
| HTT | 4049 | mU.mA.G.mU.mC.G.A.mG.A.A.mC.mC.A#mA#mA.tegChol | PmU.fU.fU.G.G.fU.fU.fC.fU.fC.G.A.fC.fU#A#A#mA#G#fC#A | yes | yes | 41.2 | 217.8 | N/A |
| HTT | 5301 | mA.mG.mU.A.mC.mU.mU.mC.A.A.mC.G.mC#mU#mA.tegChol | PmU.A.G.fC.G.fU.fU.G.A.mA.G.fU.A.fC#fU#G#fU#fC#fC#C |  |  | 36.6 | 230.2 | 0.081 |
| HTT | 6016 | mU.mU.mC.A.G.mU.mC.mU.mC.G.mU.mU.G#mU#mA.tegChol | PmU.A.fC.A.A.fC.G.A.mG.A.fC.fU.G.A#mA#fU#fU#G#fC#C |  | yes | 26.4 | 147.9 | N/A |
| HTT | 6579 | mC.mU.A.G.mC.mU.mC.mC.A.mU.G.mC.mU#mU#mA.tegChol | PmU.A.A.mG.fC.A.fU.G.G.mA.G.fC.fU.A#G#fC#A#G#mG#C |  | yes | 28.3 | 89.8 | 0.055 |
| HTT | 8603 | mC.mU.G.mC.G.mU.G.A.A.mC.A.mU.mU#mC#mA.tegChol | PmU.G.A.mA.fU.G.fU.fU.fC.A.fC.G.fC.A#G#fU#G#G#mG#C | yes | yes | 40.0 | 236.1 | N/A |
| HTT | 10125 | mC.mU.mC.A.G.G.A.mU.mU.mU.A.A.A#mA#mA.tegChol | PmU.fU.fU.fU.fU.A.A.mA.fU.fC.fC.fU.G.A#mG#A#A#mG#A#A |  | yes | 31.1 | 158.7 | 0.059 |
| HTT | 10146 | mA.mU.A.mU.mC.A.G.mU.A.A.A.G.A#mG#mA.tegChol | PmU.fC.fU.fC.fU.fU.fU.A.fC.fU.G.A.fU.A#fU#A#A#fU#fU#A | yes | yes | 25.9 | 217.7 | 0.05 |
| HTT | 10150 | mC.mA.G.mU.A.A.A.mG.A.G.A.mU.mU#mA#mA.tegChol | PmU.fU.A.A.fU.fC.fU.fC.fU.fU.fU.A.fC.fU#G#A#fU#A#fU#A | yes | yes | 28.6 | 82.2 | 0.004 |
| HTT | 424 | mC.mA.G.mC.mU.A.mC.mC.A.A.G.A.A#mA#mA.tegChol | PmU.fU.fU.fU.fC.fU.fU.G.G.fU.A.G.fC.fU#G#A#mA#A#G#U |  | yes | 67.4 | N/A | N/A |
| HTT | 456 | mC.mU.G.A.mC.A.A.mU.A.mU.G.mU.G#mA#mA.tegChol | PmU.fU.fC.A.fC.A.fU.A.fU.fU.G.fU.fC.A#G#mA#fC#A#A#U |  | yes | 51.5 | N/A | N/A |
| HTT | 522 | mG.mG.mC.A.mU.mC.G.mC.mU.A.mU.G.G#mA#mA.tegChol | PmU.fU.fC.fC.A.fU.A.G.fC.G.A.fU.G.fC#fC#fC#A#G#mA#A |  | yes | 68.2 | N/A | N/A |
| HTT | 527 | mC.mG.mC.mU.A.mU.G.G.mA.A.mC.mU.mU#mU#mA.tegChol | PmU.A.A.mA.G.fU.fU.fC.fC.A.fU.A.G.fC#G#A#fU#G#fC#C |  | yes | 45.5 | N/A | N/A |
| HTT | 878 | mU.mG.A.mC.A.A.mU.G.A.mA.A.mU.mU#mA#mA.tegChol | PmU.fU.A.A.fU.fU.fU.fC.A.fU.fU.G.fU.fC#A#fU#fU#fU#G#C |  | yes | 64.8 | N/A | N/A |
| HTT | 879 | mG.mA.mC.A.A.mU.G.A.mA.A.mU.mU.A#mA#mA.tegChol | PmU.fU.fU.A.A.fU.fU.fU.fC.A.fU.fU.G.fU#fC#A#fU#fU#fU#G |  | yes | 51.5 | N/A | N/A |
| HTT | 908 | mC.mU.mU.mC.A.mU.A.G.mC.G.A.A.mC#mC#mA.tegChol | PmU.G.G.fU.fU.fC.G.fC.fU.A.fU.G.A.mA#G#G#fC#fC#fU#U |  | yes | 99.6 | N/A | N/A |
| HTT | 1024 | mA.mU.G.mU.G.mC.mU.mC.mU.mU.A.G.G#mC#mA.tegChol | PmU.G.fC.fC.fU.A.A.mG.A.G.fC.A.fC.A#fU#fU#fU#A#G#U |  | yes | 52.9 | N/A | N/A |
| HTT | 1165 | mU.mG.A.mC.A.A.G.mG.A.A.mA.G.A#mA#mA.tegChol | PmU.fU.fU.fC.fU.fU.fU.fC.fC.fU.fU.G.fU.fC#A#fC#fU#fC#fC#G |  |  | 77.0 | N/A | N/A |
| HTT | 1207 | mU.mU.G.mU.mC.mC.A.G.G.mU.mU.mU.A#mU#mA.tegChol | PmU.A.fU.A.A.mA.fC.fC.fU.G.G.mA.fC.A#A#mG#fC#fU#G#C | yes | yes | 109.5 | N/A | N/A |
| HTT | 1212 | mC.mA.G.G.mU.mU.mU.A.mU.G.A.A.mC#mU#mA.tegChol | PmU.A.G.fU.fU.fC.A.fU.A.A.mA.fC.fC.fU#G#G#mA#fC#A#A | yes | yes | 74.9 | N/A | N/A |
| HTT | 1217 | mU.mU.A.mU.G.A.A.mC.mU.G.A.mC.G#mU#mA.tegChol | PmU.A.fC.G.fU.fC.A.G.fU.fU.fC.A.fU.A#A#mA#fC#fC#fU#G |  | yes | 104.0 | N/A | N/A |
| HTT | 1220 | mU.mG.A.A.mC.mU.G.A.mC.G.mU.mU.A#mC#mA.tegChol | PmU.G.fU.A.A.fC.G.fU.fC.A.G.fU.fU.fC#A#fU#A#A#mA#C |  | yes | 83.9 | N/A | N/A |
| HTT | 1223 | mA.mC.mU.G.A.mC.G.mU.mU.A.mC.A.mU#mC#mA.tegChol | PmU.G.A.fU.G.fU.A.A.fC.G.fU.fC.A.G#fU#fU#fC#A#fU#A |  | yes | 92.2 | N/A | N/A |
| HTT | 1227 | mA.mC.G.mU.mU.A.mC.A.mU.mC.A.mU.A#mC#mA.tegChol | PmU.G.fU.A.fU.G.A.fU.G.fU.A.A.fC.G#fU#fC#A#G#fU#U |  | yes | 81.4 | N/A | N/A |
| HTT | 1229 | mG.mU.mU.A.mC.A.mU.mC.A.mU.A.mC.A#mC#mA.tegChol | PmU.G.fU.G.fU.A.fU.G.A.fU.G.fU.A.A#fC#G#fU#fC#A#G |  | yes | 82.2 | N/A | N/A |
| HTT | 1260 | mG.mU.mU.G.mU.G.A.mC.mC.G.mG.A.G#mC#mA.tegChol | PmU.G.fC.fU.fC.fC.G.G.fU.fC.A.fC.A.A#fC#A#fU#fU#G#U |  | yes | 108.4 | N/A | N/A |
| HTT | 1403 | mU.mA.mU.mU.G.mU.G.G.A.A.mC.mU.mU#mA#mA.tegChol | PmU.fU.A.A.mG.fU.fU.fC.fC.A.fC.A.A.fU#A#fC#fU#fC#fC#C |  | yes | 138.6 | N/A | N/A |
| HTT | 1470 | mA.mA.A.G.mU.G.mC.mU.mC.mU.mU.A.G#mG#mA.tegChol | PmU.fC.fC.fU.A.A.mG.A.G.fC.A.fC.fU.fU#fU#G#fC#fC#fU#U | yes | yes | 85.6 | N/A | N/A |
| HTT | 1901 | mU.mA.mC.mC.G.A.mC.A.A.mC.mC.A.G#mU#mA.tegChol | PmU.A.fC.fU.G.G.fU.fU.G.fU.fC.G.G.fU#A#fC#fC#G#fU#C |  | yes | 81.4 | N/A | N/A |
| HTT | 1903 | mC.mC.G.A.mC.A.A.mC.mC.A.G.mU.A#mU#mA.tegChol | PmU.A.fU.A.fC.fU.G.G.fU.fU.G.fU.fC.G#G#fU#A#fC#fC#G |  | yes | 72.7 | N/A | N/A |
| HTT | 2411 | mC.mU.A.mC.A.mU.mC.G.A.mU.mC.A.mU#mG#mA.tegChol | PmU.fC.A.fU.G.A.fU.fC.G.A.fU.G.fU.A#G#fU#fU#fC#A#A |  | yes | 53.0 | N/A | N/A |
| HTT | 2412 | mU.mA.mC.A.mU.mC.G.A.mU.mC.A.mU.G#mG#mA.tegChol | PmU.fC.fC.A.fU.G.A.fU.fC.G.A.fU.G.fU#A#G#fU#fU#fC#A |  | yes | 57.1 | N/A | N/A |
| HTT | 2865 | mG.mU.G.mC.mU.mC.A.A.mU.A.A.mU.G#mU#mA.tegChol | PmU.A.fC.A.fU.fU.A.fU.fU.G.A.mG.fC.A#fC#fU#fC#G#fU#U |  | yes | 83.1 | N/A | N/A |
| HTT | 3801 | mG.mU.mU.A.mC.A.A.mC.A.A.G.mU.A#mA#mA.tegChol | PmU.fU.fU.A.fC.fU.fU.G.fU.fU.G.fU.A.A#fC#A#G#mG#A#C |  | yes | 48.9 | N/A | N/A |
| HTT | 4040 | mA.mU.mC.mC.mU.G.mC.mU.mU.mU.A.G.mU#mC#mA.tegChol | PmU.G.A.fC.fU.A.A.mA.G.fC.A.G.mG.A#fU#fU#fU#fC#A#G | yes | yes | 56.2 | N/A | N/A |
| HTT | 4048 | mU.mU.A.G.mU.mC.G.A.mG.A.A.mC.mC#mA#mA.tegChol | PmU.fU.G.G.fU.fU.fC.fU.fC.G.A.fC.fU.A#A#mA#G#fC#A#G | yes | yes | 72.2 | N/A | N/A |
| HTT | 4052 | mU.mC.G.A.mG.A.A.mC.mC.A.A.mU.G#mA#mA.tegChol | PmU.fU.fC.A.fU.fU.G.G.fU.fU.fC.fU.fC.G#A#fC#fU#A#A#A | yes | yes | 90.8 | N/A | N/A |
| HTT | 4055 | mA.mG.A.A.mC.mC.A.A.mU.G.A.mU.G#mG#mA.tegChol | PmU.fC.fC.A.fU.fC.A.fU.fU.G.G.fU.fU.fC#fU#fC#G#A#fC#U | yes | yes | 37.2 | N/A | N/A |
| HTT | 4083 | mC.mA.A.mC.A.A.mU.mU.G.mU.mU.G.A#mA#mA.tegChol | PmU.fU.fU.fC.A.A.fC.A.A.fU.fU.G.fU.fU#G#A#mA#fC#A#C |  | yes | 91.7 | N/A | N/A |
| HTT | 4275 | mA.mA.mC.A.mU.G.G.mU.G.mC.A.G.G#mC#mA.tegChol | PmU.G.fC.fC.fU.G.fC.A.fC.fC.A.fU.G.fU#fU#fC#fC#fU#fC#A | yes | yes | 77.2 | N/A | N/A |
| HTT | 4372 | mC.mA.A.A.G.A.A.mC.mC.G.mU.G.mC#mA#mA.tegChol | PmU.fU.G.fC.A.fC.G.G.fU.fU.fC.fU.fU.fU#G#fU#G#A#fC#A |  | yes | 44.5 | N/A | N/A |
| HTT | 4374 | mA.mA.G.A.A.mC.mC.G.mU.G.mC.A.G#mA#mA.tegChol | PmU.fU.fC.fU.G.fC.A.fC.G.G.fU.fU.fC.fU#fU#fU#G#fU#G#A |  | yes | 97.5 | N/A | N/A |
| HTT | 4376 | mG.mA.A.mC.mC.G.mU.G.mC.A.G.A.mU#mA#mA.tegChol | PmU.fU.A.fU.fC.fU.G.fC.A.fC.G.G.fU.fU#fC#fU#fU#fU#G#U |  | yes | 64.1 | N/A | N/A |
| HTT | 4425 | mC.mC.mU.mC.mU.mU.G.mU.mU.A.mU.A.A#mA#mA.tegChol | PmU.fU.fU.fU.A.fU.A.A.fC.A.A.mG.A.G#mG#fU#fU#fC#A#A | yes | yes | 44.6 | N/A | N/A |
| HTT | 4562 | mU.mG.G.mC.mU.mU.mU.G.mU.A.mU.mU.G#mA#mA.tegChol | PmU.fU.fC.A.A.fU.A.fC.A.A.mA.G.fC.fC#A#A#fU#A#A#A |  | yes | 102.1 | N/A | N/A |
| HTT | 4692 | mG.mG.A.A.mU.mU.mC.mC.mU.A.A.A.A#mU#mA.tegChol | PmU.A.fU.fU.fU.fU.A.G.mG.A.A.fU.fU.fC#fC#A#A#fU#G#A | yes | yes | 53.8 | N/A | N/A |
| HTT | 4721 | mU.mG.G.mC.A.mU.mC.A.mU.G.G.mC.mC#mA#mA.tegChol | PmU.fU.G.G.fC.fC.A.fU.G.A.fU.G.fC.fC#A#fU#fC#A#fC#A |  | yes | 124.2 | N/A | N/A |
| HTT | 5200 | mC.mC.mC.A.G.mU.mC.A.A.mC.mU.G.A#mA#mA.tegChol | PmU.fU.fU.fC.A.G.fU.fU.G.A.fC.fU.G.G#mG#A#A#mA#fU#C |  | yes | 43.8 | N/A | N/A |
| HTT | 5443 | mA.mG.mC.A.G.mC.A.A.mC.A.mU.A.mC#mU#mA.tegChol | PmU.A.G.fU.A.fU.G.fU.fU.G.fC.fU.G.fC#fU#fC#A#fC#fU#C |  | yes | 48.9 | N/A | N/A |
| HTT | 5515 | mG.mA.A.mU.G.mU.mU.mC.mC.G.G.A.G#mA#mA.tegChol | PmU.fU.fC.fU.fC.fC.G.G.mA.A.fC.A.fU.fU#fC#fC#A#G#mA#C | yes | yes | 62.0 | N/A | N/A |
| HTT | 8609 | mG.mA.A.mC.A.mU.mU.mC.A.mC.A.G.mC#mC#mA.tegChol | PmU.G.G.fC.fU.G.fU.G.A.mA.fU.G.fU.fU#fC#A#fC#G#fC#A | yes | yes | 47.4 | N/A | N/A |
| HTT | 10130 | mG.mA.mU.mU.mU.A.A.A.A.mU.mU.mU.A#mA#mA.tegChol | PmU.fU.fU.A.A.mA.fU.fU.fU.fU.A.A.mA.fU#fC#fC#fU#G#A#G |  | yes | 49.6 | N/A | N/A |
| HTT | 10134 | mU.mA.A.A.A.mU.mU.mU.A.A.mU.mU.A#mU#mA.tegChol | PmU.A.fU.A.A.fU.fU.A.A.mA.fU.fU.fU.fU#A#A#mA#fU#fC#C | yes | yes | 113.7 | N/A | N/A |
| HTT | 10142 | mA.mA.mU.mU.A.mU.A.mU.mC.A.G.mU.A#mA#mA.tegChol | PmU.fU.fU.A.fC.fU.G.A.fU.A.fU.A.A.fU#fU#A#A#mA#fU#U | yes | yes | 78.0 | N/A | N/A |
| HTT | 10169 | mA.mA.mC.G.mU.A.A.mC.mU.mC.mU.mU.mU#mC#mA.tegChol | PmU.G.A.mA.A.G.mA.G.fU.fU.A.fC.G.fU#fU#A#A#mA#A#U |  | yes | 69.0 | N/A | N/A |
| HTT | 10182 | mC.mU.A.mU.G.mC.mC.mC.G.mU.G.mU.A#mA#mA.tegChol | PmU.fU.fU.A.fC.A.fC.G.G.mG.fC.A.fU.A#G#mA#A#A#mG#A | yes | yes | 100.1 | N/A | N/A |
| HTT | 10186 | mG.mC.mC.mC.G.mU.G.mU.A.A.A.G.mU#mA#mA.tegChol | PmU.fU.A.fC.fU.fU.fU.A.fC.A.fC.G.G.mG#fC#A#fU#A#G#A |  | yes | 83.5 | N/A | N/A |
| HTT | 10809 | mA.mG.mU.mC.A.G.G.A.G.A.G.mU.G#mC#mA.tegChol | PmU.G.fC.A.fC.fU.fC.fU.fC.fC.fU.G.A.fC#fU#A#A#mA#A#G |  |  | 101.7 | N/A | N/A |
| HTT | 11116 | mU.mG.G.G.mU.A.mU.mU.G.A.A.mU.G#mU#mA.tegChol | PmU.A.fC.A.fU.fU.fC.A.A.fU.A.fC.fC.fC#A#A#mA#A#fC#A |  |  | 90.0 | N/A | N/A |
| HTT | 11129 | mU.mG.G.mU.A.A.G.mU.G.G.A.G.G#mA#mA.tegChol | PmU.fU.fC.fC.fU.fC.fC.A.fC.fU.fU.A.fC.fC#A#fC#A#fU#fU#C |  |  | 105.9 | N/A | N/A |
| HTT | 11134 | mA.mG.mU.G.G.A.G.G.A.A.A.mU.G#mU#mA.tegChol | PmU.A.fC.A.fU.fU.fU.fC.fC.fU.fC.fC.A.fC#fU#fU#A#fC#fC#A |  |  | 85.1 | N/A | N/A |
| HTT | 11147 | mU.mU.G.G.A.A.mC.mU.mC.mU.G.mU.G#mC#mA.tegChol | PmU.G.fC.A.fC.A.G.mA.G.fU.fU.fC.fC.A#A#fC#A#fU#fU#U |  |  | 109.9 | N/A | N/A |
| HTT | 11412 | mU.mG.A.G.G.mA.G.G.mC.mC.mC.mU.mU#mA#mA.tegChol | PmU.fU.A.A.mG.G.G.fC.fC.fU.fC.fC.fU.fC#A#A#mA#fC#A#U |  |  | 122.0 | N/A | N/A |
| HTT | 11426 | mA.mG.G.G.A.A.G.mC.mU.A.mC.mU.G#mA#mA.tegChol | PmU.fU.fC.A.G.fU.A.G.fC.fU.fU.fC.fC.fC#fU#fU#A#A#mG#G | yes |  | 106.3 | N/A | N/A |
| HTT | 11443 | mA.mU.A.A.mC.A.mC.G.mU.A.A.G.A#mA#mA.tegChol | PmU.fU.fU.fC.fU.fU.A.fC.G.fU.G.fU.fU.A#fU#A#A#fU#fU#C |  |  | 91.7 | N/A | N/A |
| HTT | 11659 | mU.mA.mC.A.mU.mU.mU.G.mU.A.A.G.A#mA#mA.tegChol | PmU.fU.fU.fC.fU.fU.A.fC.A.A.mA.fU.G.fU#A#A#mA#fC#A#U |  |  | 80.7 | N/A | N/A |
| HTT | 11666 | mG.mU.A.A.G.mA.A.A.mU.A.A.mC.A#mC#mA.tegChol | PmU.G.fU.G.fU.fU.A.fU.fU.fU.fC.fU.fU.A#fC#A#A#mA#fU#G |  |  | 98.5 | N/A | N/A |
| HTT | 11677 | mC.mA.mC.mU.G.mU.G.A.A.mU.G.mU.A#mA#mA.tegChol | PmU.fU.fU.A.fC.A.fU.fU.fC.A.fC.A.G.fU#G#fU#fU#A#fU#U |  |  | 87.8 | N/A | N/A |
| HTT | 11863 | mG.mA.G.mC.mU.mC.A.mU.mU.A.G.mU.A#mA#mA.tegChol | PmU.fU.fU.A.fC.fU.A.A.fU.G.A.mG.fC.fU#fC#A#fU#A#fU#U |  |  | 77.4 | N/A | N/A |
| HTT | 11890 | mC.mA.mC.G.mC.A.mU.A.mU.A.mC.A.mU#mA#mA.tegChol | PmU.fU.A.fU.G.fU.A.fU.A.fU.G.fC.G.fU#G#G#mG#fU#G#A |  |  | 114.3 | N/A | N/A |
| HTT | 11927 | mG.mA.mC.A.mC.A.mU.mC.mU.A.mU.A.A#mU#mA.tegChol | PmU.A.fU.fU.A.fU.A.G.mA.fU.G.fU.G.fU#fC#fU#A#fU#A#U |  |  | 113.3 | N/A | N/A |
| HTT | 11947 | mC.mA.mC.A.mC.A.mC.mC.mU.mC.mU.mC.A#mA#mA.tegChol | PmU.fU.fU.G.A.mG.A.G.mG.fU.G.fU.G.fU#G#fU#G#fU#A#A |  |  | 99.8 | N/A | N/A |
| HTT | 12163 | mU.mA.mU.mC.A.mU.G.mU.mU.mC.mC.mU.A#mA#mA.tegChol | PmU.fU.fU.A.G.mG.A.A.fC.A.fU.G.A.fU#A#A#mA#G#fU#C |  |  | 70.7 | N/A | N/A |
| HTT | 12218 | mG.mC.A.A.A.mU.G.mU.G.A.mU.mU.A#mA#mA.tegChol | PmU.fU.fU.A.A.fU.fC.A.fC.A.fU.fU.fU.G#fC#A#A#fC#A#A |  |  | 115.3 | N/A | N/A |
| HTT | 12223 | mU.mG.mU.G.A.mU.mU.A.A.mU.mU.mU.G#mG#mA.tegChol | PmU.fC.fC.A.A.mA.fU.fU.A.A.fU.fC.A.fC#A#fU#fU#fU#G#C |  |  | 114.6 | N/A | N/A |
| HTT | 12235 | mG.mG.mU.mU.G.mU.mC.A.A.G.mU.mU.mU#mU#mA.tegChol | PmU.A.A.mA.A.fC.fU.fU.G.A.fC.A.A.fC#fC#A#A#mA#fU#U |  |  | 108.3 | N/A | N/A |
| HTT | 12279 | mU.mU.mU.mC.mC.mU.G.mC.mU.G.G.mU.A#mA#mA.tegChol | PmU.fU.fU.A.fC.fC.A.G.fC.A.G.mG.A.A#mA#A#fC#A#A#A |  |  | 83.9 | N/A | N/A |
| HTT | 12282 | mC.mC.mU.G.mC.mU.G.G.mU.A.A.mU.A#mU#mA.tegChol | PmU.A.fU.A.fU.fU.A.fC.fC.A.G.fC.A.G#mG#A#A#mA#A#C |  |  | 89.9 | N/A | N/A |
| HTT | 12297 | mG.mG.G.A.A.A.G.A.mU.mU.mU.mU.A#mA#mA.tegChol | PmU.fU.fU.A.A.mA.A.fU.fC.fU.fU.fU.fC.fC#fC#G#A#fU#A#U |  |  | 82.9 | N/A | N/A |
| HTT | 12309 | mA.mA.mU.G.A.A.A.mC.mC.A.G.G.G#mU#mA.tegChol | PmU.A.fC.fC.fC.fU.G.G.fU.fU.fU.fC.A.fU#fU#A#A#mA#A#U |  |  | 73.4 | N/A | N/A |
| HTT | 12313 | mA.mA.A.mC.mC.A.G.G.G.mU.A.G.A#mA#mA.tegChol | PmU.fU.fU.fC.fU.A.fC.fC.fC.fU.G.G.fU.fU#fU#fC#A#fU#fU#A |  |  | 89.8 | N/A | N/A |
| HTT | 12331 | mU.mU.G.G.mC.A.A.mU.G.mC.A.mC.mU#mG#mA.tegChol | PmU.fC.A.G.fU.G.fC.A.fU.fU.G.fC.fC.A#A#mA#fC#A#A#U |  |  | 109.9 | N/A | N/A |
| HTT | 13136 | mC.mA.G.mU.mU.G.mU.mU.mU.mC.mU.A.A#mG#mA.tegChol | PmU.fC.fU.fU.A.G.mA.A.A.fC.A.A.fC.fU#G#A#mG#G#G#G |  |  | 113.2 | N/A | N/A |
| HTT | 13398 | mG.mA.mC.G.A.G.A.G.A.mU.G.mU.A#mU#mA.tegChol | PmU.A.fU.A.fC.A.fU.fC.fU.fC.fU.fC.G.fU#fC#A#G#fU#fC#C |  |  | 102.1 | N/A | N/A |
| HTT | 13403 | mG.mA.G.A.mU.G.mU.A.mU.A.mU.mU.mU#mA#mA.tegChol | PmU.fU.A.A.mA.fU.A.fU.A.fC.A.fU.fC.fU#fC#fU#fC#G#fU#C |  |  | 84.1 | N/A | N/A |
| HTT | 13423 | mU.mA.A.mC.mU.G.mC.mU.G.mC.A.A.A#mC#mA.tegChol | PmU.G.fU.fU.fU.G.fC.A.G.fC.A.G.fU.fU#A#A#mA#A#A#A |  |  | 124.8 | N/A | N/A |
| HTT | 13428 | mG.mC.mU.G.mC.A.A.A.mC.A.mU.mU.G#mU#mA.tegChol | PmU.A.fC.A.A.fU.G.fU.fU.fU.G.fC.A.G#fC#A#G#fU#fU#A |  |  | 114.1 | N/A | N/A |
| HTT_um | 10150 | mA.mU.A.U.C.A.G.U.A.A.A.G.A.G.A.U.U.A.A.U.U | P.U.U.A.A.U.C.U.C.U.U.U.A.C.U.G.A.U.A.U.U.U | yes | yes | N/A | N/A | 0.013 |
| HTT_Cy3 | 10150 | Cy3-mC.mA.G.mU.A.A.A.mG.A.G.A.mU.mU#mA#mA.tegChol | PmU.fU.A.A.fU.fC.fU.fC.fU.fU.fU.A.fC.fU#G#A#fU#A#fU#A | yes | yes | 28.6 | 82.2 | 0.004 |
| PPIB | 437 | mC.mA.A.A.mU.mU.mC.mC.A.mU.mC.G.mU#mG#mA.tegChol | PmU.fC.A.fC.G.A.fU.G.G.mA.A.fU.fU.fU#G#fC#fU#G#U#U | yes | yes |  |  |  |
| NTC | N/A | mA.mC.A.A.A.mU.A.mC.G.A.mU#mU#mA.tegChol | PmU.A.A.fU.fC.G.fU.A.fU.fU.fU.GU#mC#A#A#mU#mC#A |  |  | 102.0 | N/A | N/A |
| Detailed sequence, chemical modification patterns, and efficacy of hsiRNAs. Huntingtin accession number - NM_002111 .6, PPIB accession number - NM_009693.2. Chemical modifications are designated as follows. “.” – phosphodiester bond, “#” –phosphorothioate bond, “m” – 2’-O-Methyl, “f” – 2’-Fluoro, no prefix – ribonucleotide, “P” – 5’ Phosphate, “tegChol” – tetraethylene glycol (teg)-cholesterol, um – unmodified. All sequences are homologous to human huntingtin. IC50 calculated as described in materials and methods. | | | | | | | | |
